# Validation of the PICC length prediction formula based on anteroposterior chest radiographs for bedside ultrasound-guided placement

**DOI:** 10.1371/journal.pone.0277526

**Published:** 2022-11-11

**Authors:** Youngjong Cho, Sangjoon Lee, Sung-Joon Park, Hyoung Nam Lee, Hwan Hoon Chung

**Affiliations:** 1 Department of Radiology, Gangneung Asan Hospital, University of Ulsan College of Medicine, Gangneung, Gangwon-do, South Korea; 2 Vascular Center, The Eutteum Orthopedic Surgery Hospital, Paju-si, South Korea; 3 Department of Radiology, Korea University College of Medicine, Korea University Ansan Hospital, Ansan-si, South Korea; 4 Department of Radiology, Cheonan Hospital, Soonchunhyang University College of Medicine, Cheonan-si, South Korea; Stanford University School of Medicine, UNITED STATES

## Abstract

This study aimed to validate the accuracy of the peripherally inserted central catheter (PICC) length prediction formula using only anteroposterior chest radiographs (AP-CXR) and the technical feasibility of bedside ultrasound-guided PICC placement. This study included 156 Asian adult patients who underwent bedside PICC placement at three hospitals from September 2021 to March 2022. The shortest straight-line distance from the cubital crease to the puncture point (CP) was measured first. Using the formula of a previous study, the CP + estimated PICC length (eCL) was calculated with the parameters measured on AP-CXR. The formula was as follows: 19.409 + 0.424 × (MHTD, maximal horizontal thoracic diameter) + 0.287 × (CL, clavicle length) + 0.203 × (DTV, distance of thoracic vertebrae) + (2VBUs, two vertebral body units below the carina inferior border) (if from the left, 3.063cm was added; if female, 0.997cm was subtracted). Catheters were pretrimmed according to calculated eCL prior to the procedure. Technical success was evaluated, and the validation success of catheter length prediction was classified according to the catheter tip position as follows: optimal position or suboptimal position. Technical success was achieved in 153 cases (98.1%). Evaluation of validation success revealed that the position was “optimal” in 108 cases (70.6%) and “suboptimal” in 45 cases (29.4%). There was no validation failure. There was no case where the catheter was inserted too deep as to wedge into the right atrial wall. In conclusion, the PICC could be positioned accurately using the formula based on only AP-CXR. Furthermore, this bedside procedure was technically feasible.

## Introduction

The peripherally inserted central catheter (PICC) is a procedure that is widely used by healthcare providers worldwide due to its advantages of being able to be used over the long term in critical care settings [1]. The procedure is commonly performed because it is easy to perform and has a low risk of insertion-related mechanical complications such as a local hemorrhage or pleura-pulmonary injury [2].

For the precise placement of the catheter tip at the cavoatrial junction (CAJ), this procedure is usually performed under fluoroscopic guidance. However, in actual clinical situations, there are cases where bedside PICC placement is necessary. Bedside PICC placement is useful for patients who cannot endure intra-hospital transport due to cardiopulmonary instability or when there are concerns about isolation precautions due to any infection-related contamination.

Various studies have suggested methods of estimating the optimal catheter length for bedside PICC placement without fluoroscopic guidance. Some studies used landmarks of the bone and soft tissues for prediction [3–6], whereas other studies used the patient’s height as a predictor [7–9]. However, in case of severe trauma, devices intricately intertwined with each other, or contact precautions, direct length measurement using a ruler is not possible. If a patient cannot stand upright due to unconsciousness or severe hemodynamic instability in an emergency or intensive care setting, the formula based on height also cannot be used. The chest radiographs of these patients are mainly obtained with an anteroposterior projection.

A previous study by Park et al. demonstrated that the PICC length could be predicted using anteroposterior chest radiographs (AP-CXR), and related measurement factors and formulas were described [10]; however, the validation process was not included. The objective of this retrospective study was to validate the accuracy of the length prediction formula from the previous study [10] and determine whether it is technically feasible to perform PICC placement at the bedside using only ultrasound.

## Material and methods

As this was a retrospective study, formal consent was not required. This study obtained Institutional Review Board (IRB) approvals from all three participating institutions, and the need for informed consent was waived (IRB approval number of Institution A: Korea University Ansan Hospital [2022AS0074], B: Soonchunhyang University Cheonan Hospital [2022-03-021], C: Pohang St.Mary’s Hospital [0749-220304-HR-055-01]).

### Study population

This retrospective study reviewed the medical records of 156 Asian adult patients over 18 years of age who underwent bedside PICC placement without fluoroscopic guidance at three hospitals from September 2021 to March 2022.

Based on the exclusion criteria of a previous study [10], bedside PICC placement with the length prediction formula was performed only for patients who did not have the following conditions: (1) patients without previous AP-CXR, (2) patients with unusual anatomic variations including persistent left-sided superior vena cava, severe unilateral lung volume loss, or scoliosis/kyphosis/compression fracture of the vertebrae, and (3) patients with difficulty in identifying the cubital crease due to redundant skin, burns, underweight, or severe edema.

### Bedside procedure and data acquisition

**Fig 1:**
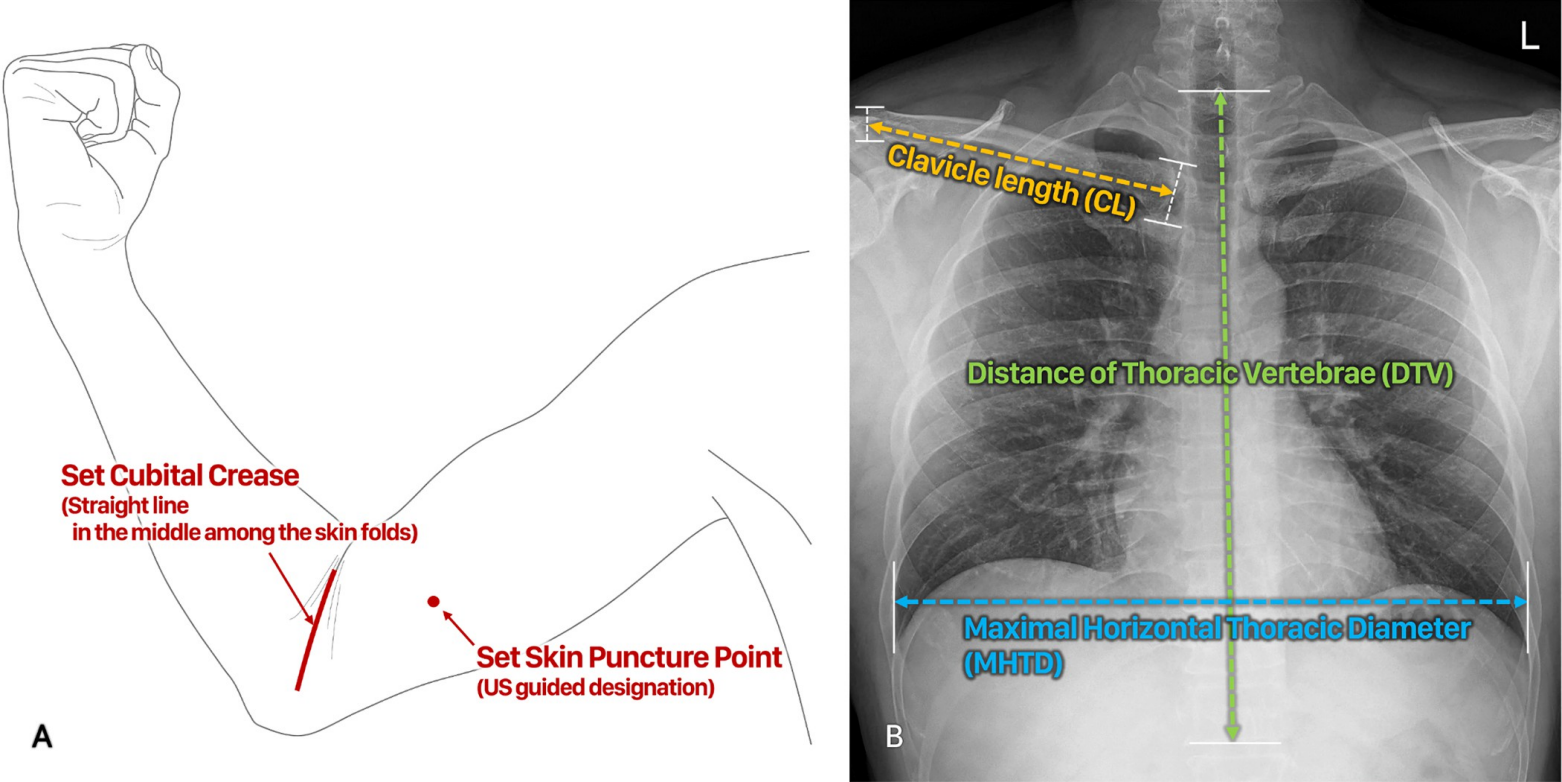
Anatomic landmarks and parameters on anteroposterior chest radiographs. (A) Designation of the cubital crease and skin puncture point. (B) Parameters measured on anteroposterior chest radiographs: (1) distance from the superior endplate of the T1 vertebra to the inferior endplate of the T12 vertebra (distance of thoracic vertebrae, DTV), (2) maximal distance between the inner edges of the ribs (maximal horizontal thoracic diameter, MHTD), (3) length between the midpoints of proximal and distal ends of the ipsilateral clavicle (clavicle length, CL).

The bedside PICC procedure was performed by four interventional radiology specialists with over 5 years of experience. After arriving at the patient’s side, first, the patient was placed in the supine position. The following steps are taken to establish the cubital crease, which was used as an anatomic landmark in the previous study [10]; the patient’s arm was abducted, and the elbow was flexed at 90°. Subsequently, the middle straight line among the skin folds was set as the cubital crease and marked. Next, the patient’s arm was straightened and rotated externally as much as possible to facilitate access to the veins of the upper arm. Ultrasound was used to locate the puncture point and vein to be accessed (Fig 1A). The basilic vein was chosen as the superficial vein; however, if the basilic vein was too small for puncturing or unidentifiable, the brachial vein was chosen as the next option. The right approach was preferred over the left approach, considering the possibility of complications, such as thrombosis or migration [11–14]. The puncture point was set at least as far away as the catheter hub length from the cubital crease so as not to be compressed when the arm was flexed. Then, the shortest straight-line distance from the cubital crease to the puncture point (CP) was measured.

All patients included in this study were lying in the intensive care unit or emergency room and could not control their breathing or change their position according to the instructions of the technologists. AP-CXR, which was used for length prediction, was selected from among the radiography previously taken, in which the patient had sufficient lung expansion and showed minimal rotated position based on the alignment of the spinous process of the spine. All pre-procedural AP-CXRs used in this study were digital radiography, and images were taken after placing an image detector (digital receiver) behind the lying patient’s back. Due to the AP-CXR device design and shooting method, it is not possible to keep source-image distance (SID) constant. However, the X-ray tube is usually raised enough to include the entire torso of the patient in the field. In most cases, it is taken at the maximum height, and considering the height of the bed, the SID is estimated to be about 100~110cm in the supine position. We also selected the most recent AP-CXR as possible.

The following parameters of the formula were measured on AP-CXR: (1) distance from the superior endplate of the T1 vertebra to the inferior endplate of the T12 vertebra (distance of thoracic vertebrae, DTV), (2) maximal distance between the inner edges of the ribs (maximal horizontal thoracic diameter, MHTD), (3) length between the midpoints of proximal and distal ends of the ipsilateral clavicle (clavicle length, CL) (Fig 1B), and (4) two vertebral body units (2VBUs) below the carina inferior border. One VBU was defined as the distance unit between the superior endplate of one vertebra to the superior endplate of the next, with the intervertebral disk included. Using the formula from the previous study [10], the CP + estimated PICC length (eCL) can be calculated. The formula was as follows:

(CP+eCL,cm)=19.409+0.424×(MHTD,cm)+0.287×(CL,cm)+0.203×(DTV,cm)+(2VBUs,cm)

If approaching from the left, add 3.063 cm; if female, subtract 0.997 cm.

**Fig 2:**
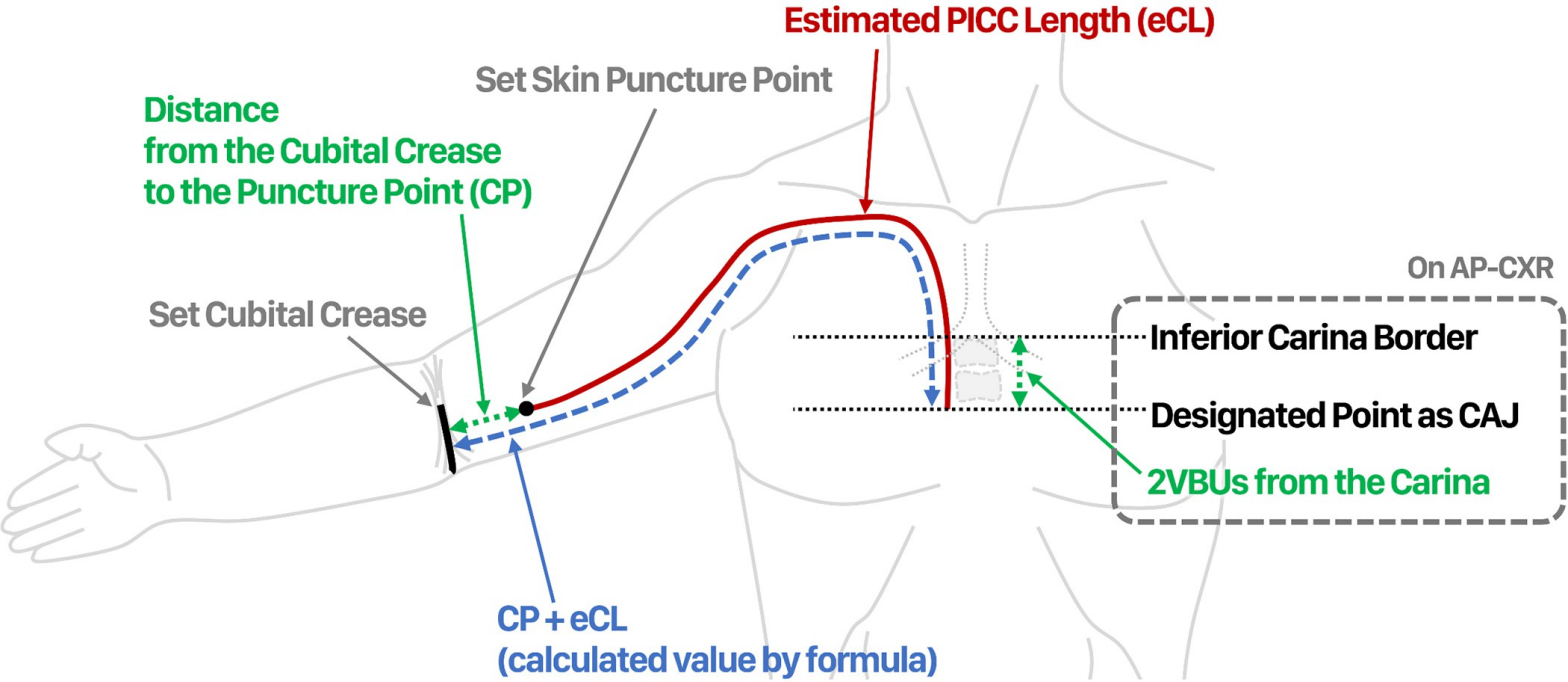
Schematic illustration of parameters. Calculation of the distance from the cubital crease to the puncture point (CP) + estimated PICC length (eCL) using the formula of Park et al. (CP + eCL, cm) = 19.409 + 0.424 × (MHTD, maximal horizontal thoracic diameter) + 0.287 × (CL, clavicle length) + 0.203 × (DTV, distance of thoracic vertebrae) + (2VBUs, two vertebral body units below the carina inferior border). If approaching from the left, add 3.063 cm; if female, subtract 0.997 cm. Catheters were pretrimmed according to the eCL prior to the procedure.

When arriving at the patient’s bedside, the CP was measured first as mentioned above, and the eCL was obtained by subtracting CP from the previously calculated CP + eCL value. Catheters (5-Fr, Power PICC; BD, Salt Lake City, UT, USA/5-Fr, Turbo-Ject PICC Set; Cook Medical, Bloomington, IN, USA) were pretrimmed according to the calculated eCL prior to the procedure (Fig 2).

**Fig 3:**
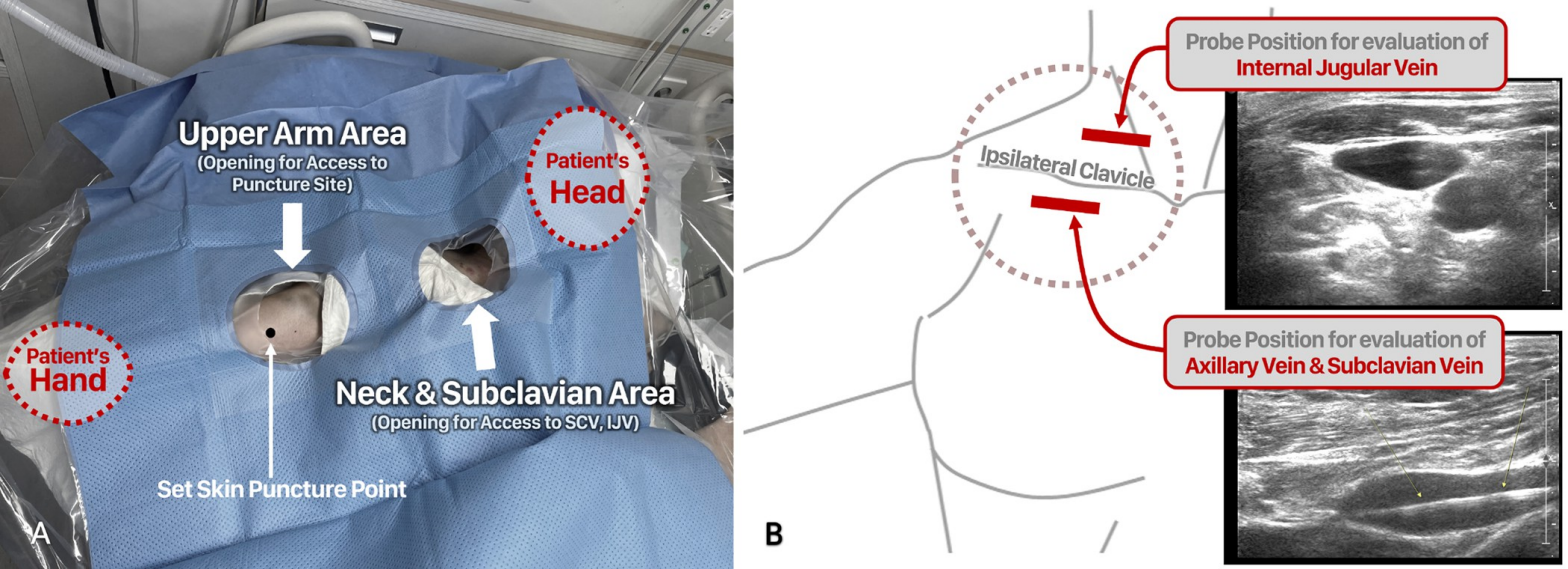
Procedure site of the PICC insertion at the bedside under ultrasound guidance. (A) Disposable drapes with two holes were used to allow the ultrasound probe to access both the puncture site of the upper arm and the neck and subclavian areas. (B) An ultrasonic probe was placed in the subclavian area to ensure that the guidewire was not twisted in the axillary vein and was advanced smoothly as a single strand. Additionally, the ipsilateral internal jugular vein was assessed to ensure that the guidewire was not inserted in the cranial direction.

The skin was prepared for the procedure in a sterile manner using chlorhexidine-alcohol. Disposable drapes with two holes (Dowoo Angiography Pack; KM Healthcare, Guri, South Korea) were used to allow the ultrasound probe to access both the puncture site of the upper arm and the neck and subclavian areas (Fig 3A). Under local anesthesia, a 21-gauge micropuncture needle was used to puncture the vein at the previously set puncture point under ultrasound guidance. The micro-guidewire was introduced in the antegrade direction. If it was judged that the guidewire more than the eCL has been inserted, the proper entry of the guidewire in the caudal direction was confirmed by ultrasound. Using an ultrasonic probe placed in the subclavian area, it was determined that the guidewire was not twisted in the axillary vein and was advanced smoothly as a single strand. Additionally, the ipsilateral internal jugular vein (IJV) was assessed by ultrasound to ensure that the guidewire was not inserted in the cranial direction (Fig 3B). If the guidewire is judged to have entered the cranial direction incorrectly, first, the guidewire was sufficiently retracted to the level of the subclavian vein or axillary vein. Then, re-entry was attempted by rotating it clockwise and counterclockwise again. Nevertheless, in case of repeatedly entering the crinal direction, it was tried again after shaping the end of the wire.

### Clinical data acquisition

Using the electronic medical records of each institute, the authors (BLINDED-FOR-REVIEW) collected and recorded the clinical information of the patients, such as sex, age, and reason for bedside procedure. In addition, detailed information on the target vein approached during the procedure was collected, such as the side (left, right), and punctured vessel name (basilic, brachial vein). In each case, before using the PICC, an AP-CXR was taken to confirm the catheter tip location and to determine whether the procedure was technically successful.

### Evaluation

**Fig 4:**
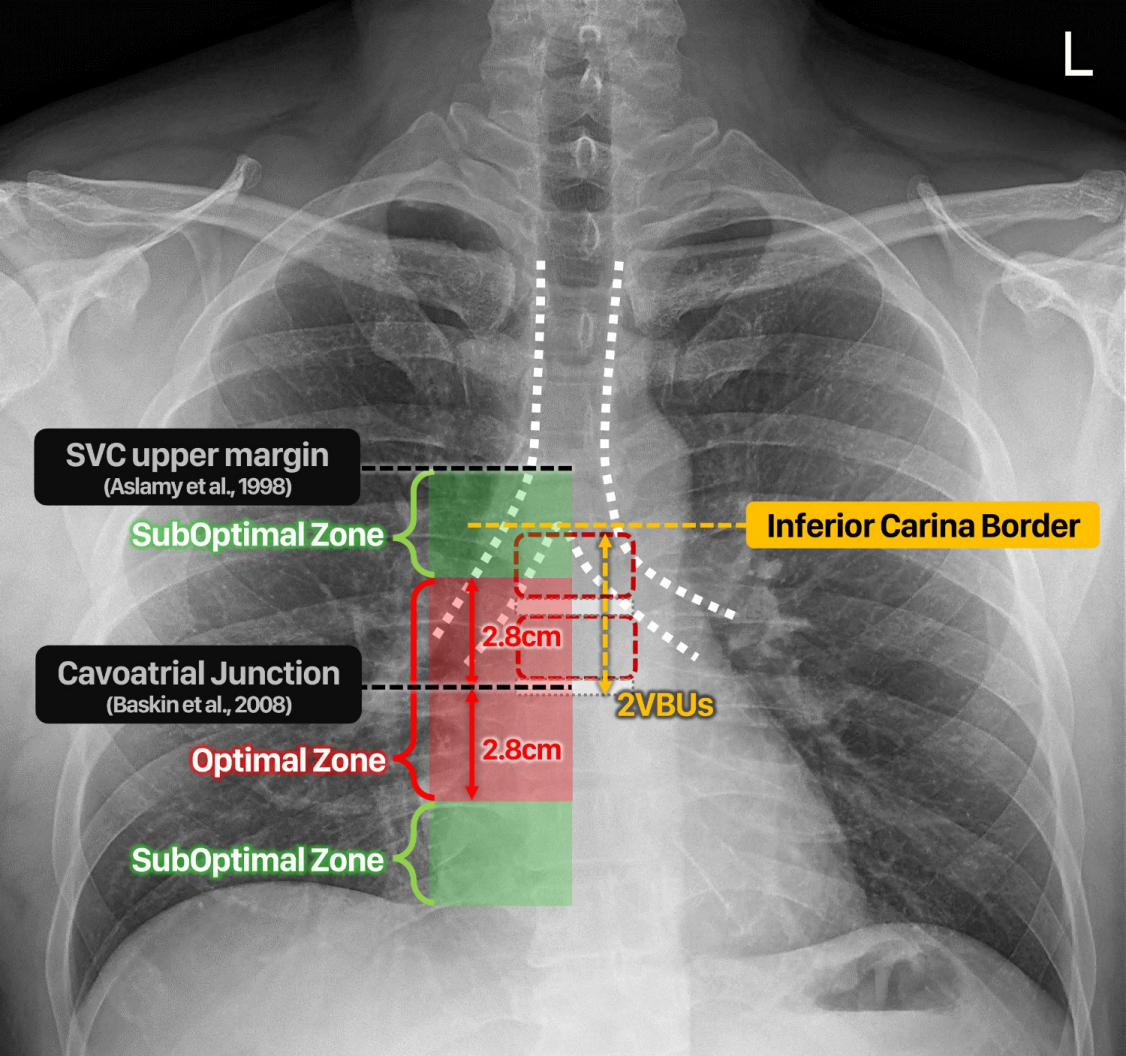
Validation success of catheter length prediction. The results were classified as optimal or suboptimal. In the "optimal" position, the tip of the catheter was located in the range of approximately 2.80 cm above and below the designated cavoatrial junction on anteroposterior chest radiographs. In the "suboptimal" position, the catheter tip was positioned in the superior vena cava (SVC) zone (below the designated upper margin of the SVC) or right atrium without wedging into the atrial wall.

The technical success and validation success of catheter length prediction was evaluated by post-procedural AP-CXR. Both of the following were required to define technical success: 1) the catheter tip must face downward in the caudal direction, and 2) the catheter tip must not be in the opposite brachiocephalic vein or azygos vein. The validation success of catheter length prediction was evaluated according to the catheter tip position on post-procedural AP-CXR for cases in which technical success was achieved, and the results were classified as optimal or suboptimal (Fig 4). In the “optimal” position, the tip of the catheter was located in the range of approximately 2.80 cm above and below the designated CAJ on AP-CXR. This range was set based on the 95% confidence level using the standard error reported in the previous study [10]. In the “suboptimal” position, the catheter tip was positioned in the superior vena cava (SVC) zone (below the designated upper margin of the SVC) or right atrium (RA) without wedging into the atrial wall. Wedging into the atrial wall refers to the degree to which the shape of the catheter itself is bent on post-procedural AP-CXR, or the aspiration of blood or fluid injection is not smooth. If the catheter tip was located at any other position, it was defined as a validation failure.

The location of the anatomic landmark on AP-CXR was defined with reference to previously published studies. The CAJ on AP-CXR was designated as two VBUs below the inferior carina border in accordance with a previous study by Baskin et al. [15]. The upper margin of the SVC was designated at the right tracheobronchial angle on AP-CXR in accordance with a previous study by Aslamy et al. [16].

The complication was evaluated in accordance with the Society of Interventional Radiology (SIR) Classification System for Complications by Outcome [17].

## Results

### Patient characteristics

The descriptive statistics of the patients and parameters measured on pre-procedural AP-CXR are listed in Table 1. The mean age of the 156 patients was 68.51 ± 14.03 years (male gender, n = 87, 56.1%), and the right approach was attempted in 140 cases (89.7%). The most frequent reason for requesting the bedside procedure was hemodynamic or respiratory instability, which accounted for 82.7% of the patients. Among them, a mechanical ventilator or extracorporeal membrane oxygenation (ECMO) was used in 35 and 9 cases, respectively. There were 52 cases of infectious disease precaution issues, accounting for 33.3% of the total patients, and 39 of them were COVID-19 patients in the isolation ward. The bedside procedure was performed for two or more combined reasons in 26 (16.7%) of 156 cases.

**Table 1:** Patient characteristics and variables measured on AP-CXR.

	Total, n = 156
**Age (years, mean ± SD, range)**	68.51 ± 14.03 (21~93)
**Sex (n, %)**	
Male	87 (55.8%)
Female	69 (44.2%)
**Approach side (n, %)**	
Right	140 (89.7%)
Left	16 (10.3%)
**Punctured vein (n, %)**	
Basilic vein	83 (53.2%)
Brachial vein	73 (46.8%)
**Reason for bedside procedure—multiple responses (n, %)**	
Hemodynamic/respiratory instability	129 (82.7%)
*Ventilator*	*35*
*ECMO*	*9*
Infectious disease precaution issues	52 (33.3%)
*Airborne/droplet precautions (COVID-19)*	*39*
*Contact precautions*	*9*
*Protective/reverse isolations*	*4*
Severe trauma	4 (2.6%)
**Parameters measured on AP-CXR (cm)**	
Maximal horizontal thoracic diameter (MHTD)	28.23 ± 2.43 (22.7 ~ 33.4)
Distance of thoracic vertebrae (DTV)	28.66 ± 2.09 (22.6 ~ 33.9)
Ipsilateral clavicle length (CL)	13.83 ± 1.43 (10.4 ~ 17.7)
Two vertebral body units (2VBUs)	4.68 ± 0.44 (3.3 ~ 5.8)
Estimated cubital crease to catheter tip distance (CP+eCL)	
*Right side approach*	*45*.*42 ± 2*.*18 (41*.*4 ~ 50*.*3)*
*Left side approach*	*48*.*50 ± 2*.*59 (43*.*0 ~ 51*.*3)*

*All included patients were Asian.

AP-CXR:anteroposterior chest radiographs;SD:standard deviation;ECMO:extracorporeal membrane oxygenation;COVID-19:coronavirus disease 2019;CP:distance from the cubital crease to the puncture point;eCL:estimated catheter length

### Technical success and complication

Technical success was achieved in 153 of 156 cases (98.1%, Table 2). In 1 of the 3 technical failure cases, the catheter tip was detected in the ipsilateral IJV on post-procedural AP-CXR. In this case, the patient had arteriovenous access for hemodialysis in the right arm, so right-side access was restricted. The patient had another central venous catheter (hemodialysis catheter for continuous renal replacement therapy) on the left neck. There was little space for the ultrasound probe to evaluate the IJV because the neck was very short, the puncture site of the catheter was just above the clavicle, and the hub was sutured to the neck. Therefore, the entry of the guidewire in the cranial direction could not be detected. In the other two cases, the guidewire failed to reach the central vein; in one case, it failed because of central vein obstruction detected on a subsequent radiologic examination, and in the other case, despite repeated attempts under ultrasound guidance, the guidewire eventually failed to pass and was simply curled.

**Table 2:** Technical success and validation success of catheter length prediction.

	Total	Right	Left
**Technical aspect (n, %)**			
Success	**153 (98.1%)**	**138**	**15**
Failure	**3 (1.9%)**	2	1
*Entry in the cranial direction (ipsilateral IJV)*	***1***		*1*
*Failure in passing through the SCV valve*	***1***	*1*	
*Central vein obstruction*	***1***	*1*	
**Validation success of catheter length prediction (n, %)**			
Optimal position	**108 (70.6%)**	**100**	**8**
Suboptimal position	**45 (29.4%)**	**38**	**7**
*In the SVC*	***37***	*32*	*5*
*Above the mid-RA without wedging into the RA wall*	***8***	*6*	*2*
**Validation failure of catheter length prediction (n, %)**	0 (0.0%)	0	0

IJV:internal jugular vein;SCV:subclavian vein;SVC:superior vena cava;RA:right atrium

According to the SIR guidelines, there were no major or minor complications.

### Validation success of catheter length prediction

Of the 153 technically successful cases, there was not a single case of validation failure (Table 2). Examining the catheter tip position on post-procedural AP-CXR revealed that the position was “optimal” in 108 cases (70.6%) and “suboptimal” in 45 cases (29.4%). When cases in the “suboptimal” position were analyzed separately, it was confirmed that the catheter tip was located in the SVC in 37 cases (82.2%) and that it was deeper than that in the “optimal” position in 8 cases (17.8%). There was no case where the catheter was inserted too deep as to wedge into the RA wall.

## Discussion

PICC placement is a safe and effective method of providing intravenous access to critically ill patients, as demonstrated in previous studies [1, 18]. Bedside PICC placement is a frequently performed technique and is being increasingly used in various clinical settings [19–21]. However, the most challenging aspects of a successful bedside PICC procedure are 1) determining the appropriate catheter length and 2) placing the catheter caudally from the ipsilateral subclavian vein toward the CAJ. In a retrospective review of PICC placements performed at the bedside, Trerotola et al. reported that there were around 10% of cases of catheter tip malposition [22].

The optimal catheter tip position in the SVC remains a controversial issue [23]. The United States Food and Drug Administration (USFDA) recommended that “the catheter tip should not be placed in, or be allowed to migrate, into the heart” [15, 24]. However, the KDOQI 2019 guidelines recommended that the “proper location of the central venous catheter tip is at the mid-RA to avoid vessel and right atrial trauma and consequent complications” [25]. In 1998, the National Association of Vascular Access Network suggested that the tip of the PICC should be positioned within the lower third of the SVC, close to the CAJ [26]. Furthermore, in 2000, the Infusion Nurses Society indicated in a Standards of Practice document that “central catheters should have the distal tip dwelling in the vena cava” [27]. Although there are various different viewpoints, it is generally recommended to avoid intracardiac catheter placement (as suggested by the USFDA) in order to reduce the possibility of fatal complications such as arrhythmia, or cardiac tamponade related to cardiac perforation [28–30]. Overall, based on these guidelines, it was concluded that the optimal location of the catheter tip for central venous access is around the CAJ, and more broadly, from the SVC to the mid-RA (not too deep). This study set this range as the criterion for validation success in length prediction.

As the bedside PICC procedure is performed without fluoroscopy, several methods have been proposed for determining the catheter’s length in adult patients. There have also been studies that predicted the PICC length using chest radiographs [10, 31]. The study by Ramamurthi et al. is the first study to use CXR measurements to predict the PICC length [31]. The study was conducted on pediatric patients, and the distance from the mid-humerus to the CAJ was directly measured on a radiograph. However, in adults, it is difficult to predict the PICC length through direct measurement because the CXR does not include the entire humerus in most cases. Park et al. reported a formula derived from elements measured on AP-CXR instead of direct measurement [10]. In this study, the formula by Park et al. was validated; technical success was achieved in 96.8% of cases, and there was no failure with respect to the validation success of catheter length prediction (optimal 70.2%, suboptimal 29.8%) among technically successful cases.

The malposition of a catheter tip for central venous access may result in complications such as perforation of the vascular wall, thrombus formation in the vein, catheter dysfunction, or cranial retrograde injection [32]. In this study, to minimize catheter tip malposition, the axillary, subclavian, and internal jugular veins were observed by ultrasound during the procedure. In some cases, ultrasound examination was disrupted if there was a central vein catheter, such as a hemodialysis catheter or Hickman catheter, in the ipsilateral neck or chest. However, in all other cases, the direction of the wire was examined through ultrasound before proceeding to the next step.

There have been several other efforts to determine the optimal catheter tip position for PICC placement. Electromagnetic positioning systems, such as Sherlock 3CG^TM^ Tip Confirmation System (BD), have been introduced; however, they are not always suitable for daily clinical use due to cost-effectiveness or distribution issues [33, 34]. Cho et al. reported an attempt to include portable digital radiography (DR) in bedside PICC placement [35]. The method of utilizing DR for bedside PICC placement has the advantage of being able to accurately set the length of the catheter using a guidewire intuitively. However, portable DR imaging is also a source of unnecessary radiation exposure to nearby patients and medical staff in intensive care units or emergency rooms without individual rooms. For patients with an unfavorable anatomical structure in which the guidewire easily enters the internal jugular vein rather than the CAJ direction from the subclavian vein, the movement of the guidewire must be observed in real-time to be able to push it in the correct caudal direction. Examining the direction of the guidewire through DR is a non-real-time method; thus, it can only detect events that have occurred with additional radiation exposure. The electromagnetic positioning system can also help determine the catheter tip position but is not practically helpful in correcting inaccurate guidewire entry in the cranial direction. Through the use of ultrasound in this study, the erroneous entry of the guidewire in the cranial direction could be detected in real-time without radiation exposure, and correction could be confirmed by manipulating the wire with real-time visual monitoring. Even when another catheter is already located in the IJV on the ipsilateral side, this method can be applied if the ultrasound probe can reach the neck avoiding the puncture site. In fact, in 21 cases, another catheter was already located in the ipsilateral IJV before the bedside PICC placement, and successful insertion was possible under real-time monitoring through the ultrasound in 20 cases except for one malposition case mentioned in the results section (8 CVCs, 6 temporary hemodialysis catheter, 5 permanent hemodialysis catheter, and one totally implantable venous-access port).

In this study, three hospitals performed validation using the formula with only AP-CXR for predicting the PICC length. It was found that the formula could be applied successfully without length prediction failure. Chi-square analysis of the length prediction results of the three hospitals did not show any statistically significant differences (*p* = 0.274).

This study has some limitations. First, this was a retrospective study based on existing medical records. Second, although several institutions were included in the study, the number of included cases was small for the analysis of complications or left-sided approaches. Third, the use of CXR with an anteroposterior profile to reflect actual clinical situations inevitably included unavoidable measurement errors. AP-CXRs were filmed in a limited space on the bedside, and they have a technical limitation in that it is difficult to keep the SID constant. As is known, if the SID is not kept constant, it may affect the magnification. In particular, as AP-CXR is mainly taken for patients with poor coordination or lowered consciousness, such as those in the emergency room, trauma bay, or intensive care unit, it may be taken in most cases without breathing control.

In the previous study by Park et al., for the formula, the distance from the CAJ to the catheter tip was measured by fluoroscopy, and the remaining factors were measured by AP-CXR; thus, the mixed imaging modalities may be regarded as a limitation. In addition, the length of the arm may vary for each patient; however, this was not thoroughly considered in the formula development process. In addition, when tracheal deviation occurs due to tumor, atelectasis, or lung volume loss, the horizontal factor of length is greatly affected. However, there was a limitation that length measurement using only bony landmarks did not reflect this. Further research should be conducted to clarify these issues.

## Conclusion

In conclusion, even in the absence of fluoroscopic guidance, the PICC may be positioned accurately using the pre-existing formula based on only AP-CXR. Furthermore, this bedside procedure was technically feasible in most cases.

## Supporting information

S1 Data (XLSX)
